# Spontaneous cervical epidural hematoma associated with thunderclap headache

**DOI:** 10.1007/s10194-012-0433-x

**Published:** 2012-03-16

**Authors:** Daniel Schwartz, Karthikeyan Arcot, Brian M. Grosberg, Matthew S. Robbins

**Affiliations:** Montefiore Headache Center, Albert Einstein College of Medicine, Bronx, NY USA

## Case report

An 80-year-old woman abruptly developed an explosive, severe headache in the bilateral occipital and frontal regions associated with neck pain/stiffness while sitting on her couch watching television. There was no associated loss of consciousness or awareness, nausea, vomiting, autonomic symptoms, photophobia or phonophobia. She denied any preceding neck pain or history of head or neck trauma.

Prior to this event, she rarely experienced mild, infrequent episodic headaches lasting a few hours only which were relieved by aspirin. Her medical history included diabetes, hypertension, coronary artery disease, prior cerebellar infarcts, glaucoma, and deep venous thrombosis for which she was receiving anticoagulation. Her medications included warfarin, aspirin, clopidogrel, simvastatin, valsartan, and carvedilol. She was a heavy smoker, but denied alcohol or drug use.

In the emergency department she was afebrile and had mild neck stiffness, particularly on neck flexion. Blood pressure was 168/89 mm Hg with a heart rate of 85 beats per minute. Neurological examination did not reveal papilledema or any focal, long tract, or lateralizing signs. Cranial computed tomography (CT) was negative for intracranial hemorrhage. Blood work revealed a markedly elevated INR (6.0), but CBC, electrolytes, ESR (26 mm/h), urinalysis, and serum glucose (138 mg/dL) were normal. The patient was administered 4 U of fresh frozen plasma to reverse her coagulopathy to perform an emergent lumbar puncture; during this time she underwent magnetic resonance imaging (MRI) and angiography (MRA) of the brain and neck (time-of-flight with fat-suppression), which were remarkable only for evidence of prior bilateral cerebellar and small deep left middle cerebral artery territory infarcts. Although magnetic resonance venography (MRV) was not performed, contrast-enhanced MRI revealed patency and normal enhancement of the venous sinuses.

Cerebrospinal fluid (CSF) examination demonstrated subarachnoid hemorrhage (SAH) as well as a neutrophilic pleocytosis (540 red blood cells and 8 white blood cells per high power field in tube 1, 2,100 red blood cells and 15 white blood cells per high power field in tube 4, 96 % polymorphonuclear leukocytes, glucose 100 mg/dL), with elevated CSF protein levels (72 mg/dL). Gram stain and subsequent CSF cultures revealed no causative organism. Chest X-ray did not demonstrate any infiltrate. Given the possibility of a central nervous system (CNS) infectious process, the patient was empirically treated with intravenous antibiotics (vancomycin, ceftriaxone, ampicillin) and acyclovir.

**Fig. 1:**
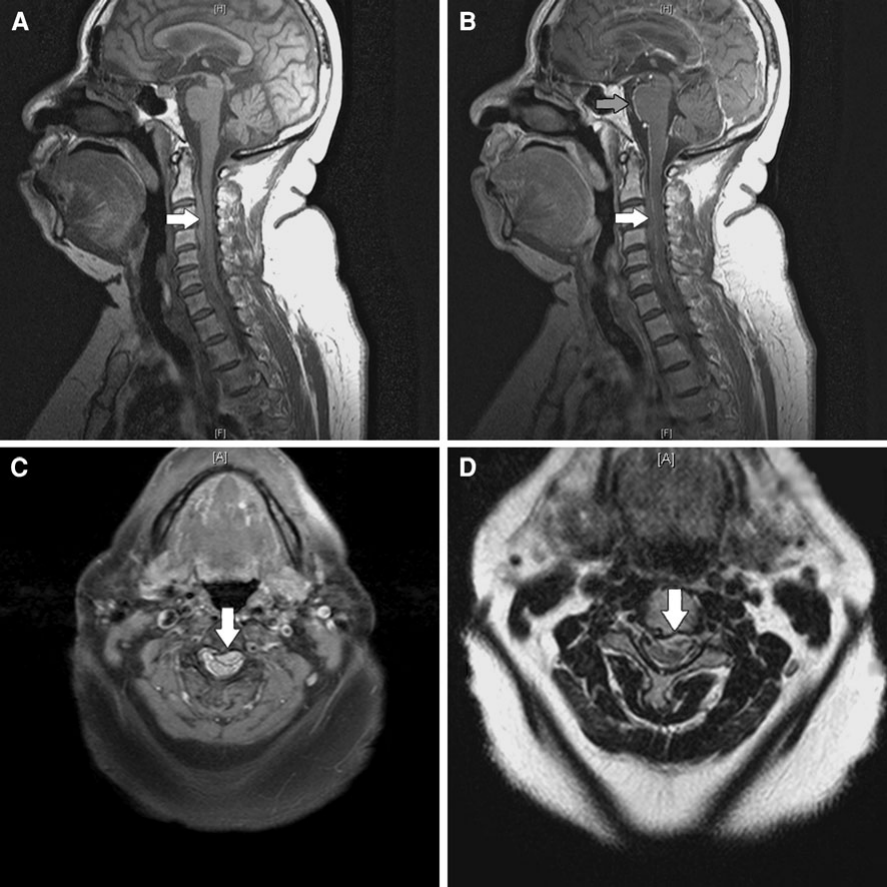
Sagittal pre (**a**) and post-contrast (**b**) T1 MRI demonstrate an epidural collection spanning from mid-C2 through T1/T2 vertebral level (*white arrow*). Axial T1 (**c**) and T2 (**d**) MRI demonstrate regions of mixed intensity within the cervical epidural collection (*white arrow*), suggestive of spontaneous spinal epidural hematoma (SSEH). A rim of contrast enhancement is seen extending from the upper cervical cord to the ventral medulla and anterior pons (**b**, *gray arrow*)

Because of the persistence of head and neck pain, her history of diabetes, and warfarin combined with dual antiplatelet agent use, a cervical epidural etiology (infectious or hemorrhagic) was suspected. Gadolinium-enhanced MRI of the cervical and thoracic spine demonstrated a heterogeneous cervical epidural collection spanning from mid-C2 through T1/T2 vertebral level, with a thin rim of contrast enhancement extending from the upper cervical cord to the anterior pons (Fig. 1). Gradient-echo sequences (not shown) revealed few areas of hypointensity, suggestive of a cervical epidural hematoma with acute elements.

The headache and neck pain resolved several days after withholding anticoagulation and treating with empiric intravenous antibiotics (vancomycin and ceftriaxone).

## Discussion

Thunderclap headache (TCH) refers to a severe and explosive headache with peak intensity at onset—as sudden and as unexpected as a ‘clap’ of thunder. Although the term is most classically used to describe the presentation of a ruptured cerebral aneurysm, there has been an increasing number of conditions reported presenting with TCH. These include SAH, an unruptured cerebral aneurysm, cerebral venous sinus thrombosis, carotid and vertebral artery dissection, infectious meningitis, spontaneous intracranial hypotension, hypertensive encephalopathy, pituitary apoplexy, retroclival hematoma, and reversible cerebral vasoconstriction syndrome [1].

While a recent report demonstrated a patient with a cervical epidural abscess presenting with cluster-like headaches, cervical epidural pathologies are not typically included in the differential diagnosis of TCH [2]. Our patient presented with TCH associated with a secondary, largely extracranial etiology—a spontaneous spinal epidural hematoma (SSEH). We considered a cervical epidural abscess in the differential diagnosis particularly because of her history of diabetes, a well-characterized risk factor for spinal epidural abscess [3]. However, her WBC and ESR were within normal limits, her abnormal CSF studies may have been nonspecific and related to a parameningeal inflammatory process, and her MRI showed clear acute hemorrhage. To the best of our knowledge, this is the first reported case of SSEH associated with a TCH presentation. Our case report was similar in both clinical presentation and in neuroimaging to the single case report of a patient presenting with TCH in the setting of a spontaneous retroclival hematoma [4]. That patient also presented with TCH, for which cerebral imaging and CSF analysis did not yield a specific etiology. Only when cervical spine MRI was performed did the diagnosis become clear.

As is the case in cervicogenic headache, the proposed mechanism underlying referred pain in our patient may involve convergence between cervical and trigeminal afferents in the trigeminocervical complex [5]. Any structure innervated by any of the upper three cervical spinal nerves, including the upper cervical synovial joints, the upper cervical muscles, and the dura mater of the upper spinal cord could be a source of headache if the central terminals of its nerve supply converged with trigeminal afferents, or with cervical afferents from the occiput [6]. Alternatively, TCH may have developed when the SSEH was complicated by local SAH in the cervical cord and lower brainstem (as supported by the hemorrhagic CSF profile), the presence of meningeal inflammation in that region, or both phenomena.

This case demonstrates that TCH may be associated with SSEH. Although numerous reports have described other responsible etiologies in TCH, cervical epidural pathologies are not typically included in the differential diagnosis. The recognition that SSEH may present with TCH has important clinical implications. Magnetic resonance imaging of the brain and cervical spine is often recommended for the evaluation of patients with an SAH and normal angiographic studies, and this case supports that approach [7]. As emphasized elsewhere, the findings in our patient underscore the importance of MR imaging of the cervical spine in excluding paraspinal cervical pathologies in patients with TCH and nondiagnostic CSF analysis, and cerebrovascular neuroimaging [4].
